# Assessing maternal nutrition policy and programmatic landscape in India: a qualitative study

**DOI:** 10.3389/fnut.2026.1831098

**Published:** 2026-10-07

**Authors:** Sukirti Chandra, Avilasha Ghosh, Himani Sharma, Shweta Khandelwal

**Affiliations:** 1 Indian Institute of Public Health, Gandhinagar, Gujarat, India; 2 Department of Humanities and Social Sciences, Indian Institute of Technology, New Delhi, India; 3 Department of Survey Research and Data Analytics, International Institute for Population Sciences, Mumbai, India; 4 Senior Public Health Nutrition Expert, Centre for Chronic Disease Control (CCDC), New Delhi, India

**Keywords:** India, maternal nutrition, policy, service delivery, women’s health

## Abstract

**Introduction:**

Maternal nutrition is fundamental to the health of mothers and children. Over the years, the Indian government introduced several programs and policies to support women’s nutrition during pregnancy and improve maternal and child health outcomes. However, significant contextual differences remain in service provisions and access across states. This study explores expert perspectives on factors that impact the implementation and reach of maternal nutrition services in India.

**Methods:**

The study was conducted in two phases. The first phase consisted of contextual mapping through desk review of policy documents, government reports, published literature, and descriptive secondary data from NFHS-4 and NFHS-5 to map maternal nutrition indicators and provide contextual evidence. In the second phase, fifteen national- and state-level experts from across policy, implementation, and research fields were purposively selected for semi-structured interviews. Following this, interviews were transcribed, coded and analyzed using a reflexive thematic analysis.

**Results:**

The desk review indicated that maternal nutrition programs in India are implemented through multiple local and centralized institutions and show substantial variation across states. State-specific initiatives to strengthen maternal nutrition were identified in 35 of the 36 Indian states and union territories. Qualitative findings complemented the desk review. Thematic analysis of expert interviews indicated that despite numerous maternal nutrition programmes in effect in India, effective implementation was constrained by three interconnected mechanisms: weak governance and health system capacity to translate policy into consistent frontline practice; fragmented monitoring systems that limit routine, data-driven course correction; and sociocultural norms that impact household decision-making and the reach and acceptability of maternal nutrition services.

**Discussion:**

Experts emphasized that strengthening implementation within existing government programmes, rather than introducing additional schemes, is central to improving maternal nutrition services and, ultimately, maternal health outcomes in India.

## Introduction

Maternal malnutrition, including underweight, anemia, and overweight, remains a major public health challenge globally and in India. According to India’s National Family Health Survey (NFHS-5) 2021, 19% of women of reproductive age are thin for their height (BMI less than 18.5 kg/m^2^), 24% are obese (BMI greater than 25 kg/m^2^), 57% are anemic (Hb levels < 12.0 g/dL), and 15% are adolescent mothers (1). This dual burden of malnutrition is driven by a complex interplay of biological, socioeconomic, and environmental factors. Maternal undernutrition is reinforced by early pregnancies, poverty, caste inequities, and gender norms, while rising availability of energy-dense, nutrient-poor foods, alongside high-sugar public food systems, continues to fuel overweight, obesity, and related non-communicable diseases (NCDs) (2, 3).

Inadequate or excessive gestational weight gain is linked to adverse birth outcomes, including low birth weight, preterm birth, and large-for-gestational-age infants (4–6). Maternal nutrition is further constrained by inadequate care provisions, cultural food taboos, gender norms, poverty, and limited nutrition knowledge (4, 7). Providing an enabling environment through enhanced prioritization and strategic investment in maternal nutrition is essential to address existing gaps. Over the years, various initiatives have been introduced and expanded by the Government of India to directly provide food or supplements specifically during pregnancy and lactation. These schemes are primarily implemented through the flagship programs of two ministries: Mission Saksham Anganwadi and Poshan 2.0, earlier known as the Integrated Child Development Services (ICDS) under the Ministry of Women and Child Development (WCD), which provides pregnant and breastfeeding women micronutrient-fortified supplements and energy-dense take-home rations (THR) which are government-provided fortified food supplements intended to provide additional energy and protein during pregnancy and lactation for home consumption and The Ministry of Health and Family Welfare (MoHFW), which offers micronutrient supplements (IFA and calcium), deworming, weight gain monitoring, and dietary counseling as part of antenatal care services for pregnant women. India’s current strategies to improve maternal diets encompass a comprehensive range of interventions, including take-home rations and hot cooked meals for pregnant and lactating women, micronutrient supplementation, food fortification, subsidized staples via the Public Distribution System, financial assistance, and dietary counseling. India also has a broad set of social protection programs that influence nutrition, both directly and indirectly, across different stages of life, as described in the Social Protection for Nutrition in India: An Atlas (8). Yet, progress remains constrained by weak delivery systems, logistical challenges, limited resources, gaps in program reach, and persistent inequities in access to food and health services among pregnant women (2).

Despite policy commitment and program priority by the Indian government to improve maternal nutrition, many proven maternal nutrition interventions are yet to reach the majority of pregnant women and young mothers in rural regions. Although the ANC registration numbers are high, data shows that only half of the pregnant women receive more than 4 visits by frontline workers, and only one-third consume more than 100 IFA tablets (9). Despite substantial policy commitment, important implementation gaps remain in maternal nutrition services in India. While existing evidence describes coverage gaps in specific interventions, there is limited qualitative evidence synthesizing the perspectives of experts involved in maternal nutrition policy, implementation, and research across India. To address this gap, we combined a descriptive review of NFHS-4 and NFHS-5 maternal nutrition indicators with a national and state-level mapping of maternal nutrition initiatives and qualitative interviews involving experts to provide a systems-level understanding of implementation challenges and opportunities for strengthening maternal nutrition service delivery and program reach in India.

## Materials and methods

### Research design

We conducted a cross-sectional qualitative study between January and June 2024 to identify key challenges in the service delivery of maternal nutrition programs in different regions of India. The study involved two phases. In Phase 1, an extensive contextual mapping desk review of secondary literature was conducted to gain an overview of the existing maternal nutrition services and programs provided by the Government of India. Our assessment included identifying and mapping the state-specific maternal nutrition interventions. Additionally, data from the National Family Health Survey (NFHS)-4 and 5 estimates were descriptively reviewed to contextualize the burden of maternal underweight and overweight in India. No independent statistical analysis of NFHS datasets was undertaken. The desk review and descriptive secondary data were intended to contextualize the qualitative findings rather than serve as an independent quantitative analysis. The corresponding descriptive figures from NFHS data are presented in Supplementary Figures 3−7.

Following the desk review, in Phase 2, using purposive sampling, we identified and contacted individuals directly involved in program management, implementation, policy, and research in maternal nutrition. Participants were selected on the criterion of having a minimum of 5 years of professional experience and active engagement with maternal nutrition programs and services in India. Stakeholders working outside the Indian context or whose primary expertise was unrelated to maternal nutrition were excluded from the study.

In phase 2, a total of 15 In-Depth Interviews (IDIs) were conducted with key stakeholders, comprising implementation experts, researchers, medical professionals, and policymakers using an interview guide (Supplementary file 2). Interviews were conducted in English, either in person or virtually, depending on logistical feasibility and participants’ preference. All sessions were conducted by trained qualitative researchers and the interviews were audio-recorded with informed consent from respondents. We supplemented our primary data with detailed field notes to ensure accuracy of data reporting, validity of findings, and the inclusion of adequate contextual details in the analysis. Each interview lasted between 30 and 75 min, and the recordings were later transcribed verbatim for further analysis. All interviews were manually transcribed by the authors. The transcripts were double-checked by the authors for accuracy. Each author independently coded the transcripts and subsequently refined the codes, resulting in the development of a structured codebook using Microsoft Excel. The finalized codebook was then used to organize codes into common and unique themes and sub-themes.

### Data analysis

We analyzed the qualitative data using a codebook-based, reflexive thematic analysis informed by Braun and Clarke’s principles (2006) (10). This framework selection was informed by the desk review undertaken by authors. In the inductive phase, the research team identified new patterns from the collected data. Using the six-phase approach proposed by Braun and Clarke, the interview transcripts were analyzed thematically, involving data familiarization, generation of initial codes and sub-codes, identifying and defining themes, reviewing recurring themes, and producing a detailed report. Coding and thematic analysis were managed manually using Microsoft Excel. These emergent codes were refined and grouped into broader themes and sub-themes that reflected systemic, structural, and individual challenges. A detailed coding sheet was developed to position each participant’s responses against key themes and sub-themes, enhancing analytical rigor and allowing for comparative analysis across stakeholder categories. Lastly, the findings were interpreted with reference to the WHO Health Systems Framework to situate the identified barriers within the broader health system context (11).

### Ethical considerations

Prior to data collection, written informed consent was obtained from all participants after explaining the purpose of the study, their voluntary participation and their right to withdraw from the interview at any time without consequences. Confidentiality and anonymity were ensured, and all personal identifiers were removed during transcription and analysis. The study protocol received ethical approval from the Institutional Ethics Committee of the Indian Institute of Public Health, Gandhinagar (IIPHG) (IEC No.: TRC/2023–24/21–17).

## Results

### Phase 1: desk review

The desk review showed that maternal nutrition programs were being implemented across states but the interventions and service delivery varied considerably. For instance, Odisha and Chhattisgarh promoted millet-based THR, Gujarat and Haryana emphasized fortified milk and double-fortified salt, Tamil Nadu and Rajasthan provide maternity benefit schemes along with nutrition kits, and Telangana and Andhra Pradesh implemented hot cooked meal models for pregnant and lactating women (Supplementary file 1). Although most of these initiatives were delivered through ICDS and the public health system, differences in design, scale, and cross-departmental convergence pointed to variations in how programs functioned in practice, thereby necessitating deeper qualitative examination.

Supplementary Figure 1 maps the key ministerial platforms through which maternal nutrition services are delivered. Supplementary Figure 2 illustrates potential entry points for strengthening integration within these existing platforms.

The descriptive analysis of NFHS-4 and NFHS-5 further highlighted the persistent dual burden of malnutrition among women in India, with both underweight and overweight remaining important public health concerns (Supplementary Figure 2). These findings provided the epidemiological context for interpreting the qualitative findings from expert interviews.

### Study participants

A total of 15 IDIs were conducted using a semi-structured interview guide with experts who had extensively worked in state and national maternal nutrition initiatives, whether in program implementation, policy, or research. As summarized in Table 1, participants represented a mix of governance levels, genders, and institutional affiliations, enabling the study to capture perspectives from decision-making, implementation, and evidence-generation contexts. These interviews yielded various perspectives that were essential for achieving the study’s objectives.

**Table 1:** Profile of the respondents in the study.

Respondents’ characteristics	*N*	Percent
Administrative level		
National	12	80.0
State	3	20.0
Gender		
Male	6	40.0
Female	9	60.0
Affiliation		
Researcher	4	26.7
NGO/partner	10	66.7
Ex-government	1	6.7
Total	15	100.0

Our analysis pointed to important system strengths, including the presence of large-scale national platforms for maternal nutrition, the commitment of frontline workers to service delivery, and the emergence of digital systems such as Family Planning Logistics Management Information System (FPLMIS) and e-Aushadhi that have begun to improve supply tracking and program oversight. Participants also highlighted growing recognition within policy circles of the need for more comprehensive and integrated approaches to women’s nutrition.

At the same time, the findings indicate several areas within the health system that could be strengthened to enhance maternal nutrition outcomes, including administration, policy, and governance, infrastructure, program monitoring, and broader socioeconomic inequalities. At the beginning of the analysis, we organized the data under eight sub-themes based on our first reading of the transcripts and codes. However, as we continued reviewing and comparing responses and codes, we realized that several of these subthemes were closely related or overlapping. The findings were synthesized into eight sub-themes under three overarching themes: (1) Policy, governance and health system capacity, (2) Information systems and Program Monitoring, and (3) Community and Sociocultural Barriers.

#### Policy, governance, and health system capacity

##### Absence of a comprehensive maternal nutrition strategy

Participants highlighted the lack of an integrated maternal nutrition strategy that simultaneously addresses undernutrition, micronutrient deficiencies, and the rising prevalence of overweight and obesity among women. Existing programs were perceived as fragmented, with insufficient state-specific implementation frameworks and limited coordination across sectors.

An expert pointed out, “*We cannot have a nutritional strategy that only focuses on minimizing the burden of obesity and overweight in Indian women. The strategy must include a comprehensive approach that addresses nutritional deficiencies while also controlling the rise of overweight and obesity*.”

Respondents further noted that routine program evaluations remain infrequent, limiting opportunities to identify implementation gaps and adapt policies based on evidence.

##### Capacity building issues

A major finding concerned inadequate competencies in nutrition counseling among frontline health workers (FLWs). Participants consistently distinguished between simply providing health messages and delivering behavior change counseling that addresses women’s concerns, dietary practices and family influences. FLWs are often unable to convey relevant messages to pregnant women, such as the importance of consuming Take Home Ration (THR), how to integrate it into daily routines, and the significance of dietary diversity and sharing THR information with immediate families. Experts highlighted an urgent need to train FLWs in nutrition counseling to enhance their confidence and capacities in delivering maternal nutrition services.

One of our respondents indicated, “*due to lack of counseling, pregnant women are not able to understand the importance of taking Take Home Ration (THR), how and why she should take it, and not share it with the whole family. Therefore, strengthened counseling can address all these issues*.”

Similarly, workers often fail to address prevalent myths, misconceptions, and fears surrounding iron and folic acid (IFA) consumption among women. Even when FLWs possess the necessary knowledge and information to address women’s concerns regarding the consumption and side-effects of IFA, they do not effectively communicate them to beneficiaries.

An experienced implementation expert said, “*The compliance with iron folic acid supplements during pregnancy is very poor. It is common among the women to say that iron causes gastritis, and it does, but a simple message like ‘take it after dinner,’ or ‘do not take it with tea, coffee, or calcium,’ can help.”*

Gaps in effective communication present a significant barrier to ensuring that women receive accurate information about IFA supplementation and other health and nutrition services.

##### Nascent presence of preconception nutrition within existing programs

Experts repeatedly identified preconception care as one of the weakest components of maternal nutrition services. Current interventions primarily begin after pregnancy confirmation, thereby missing a critical opportunity to improve women’s nutritional status before conception and reduce future maternal and neonatal risks.

Respondents recommended expanding nutrition education beyond pregnancy to include adolescent girls, newly married women and women of reproductive age through community-based platforms.

An implementation expert said, “*there is an absence of a system which is looking at nutrition counseling. A medical officer hardly gives any information on nutrition when a pregnant woman needs it. So, it is not the question of knowledge. Everybody knows about the physiological aspects of pregnancy, but whether doctors are aware of how micronutrient deficiencies can put women at risk, or if a system is in place in terms of helping women understand deficiencies and correcting it are the questions we ought to ask. The ideal time to correct all this gap in knowledge is preconception*.”

##### Frontline workforce overload and weak service delivery

Stakeholders described substantial workload pressures among Accredited Social Health Activists (ASHAs) and Anganwadi Workers (AWWs), who simultaneously implement multiple national health programs. This competing workload reduces the time available for individualized nutrition counseling, home visits and follow-up support.

A nutrition expert explained, “*If you look at the work profile of these frontline functionaries, I believe that we are stretching their focus to too many areas. There are several programs, especially health programs, and the ASHA is stretched to many programs*.”

Similarly, FLWs across most PHCs are tasked to document all the services they provide, which often introduces reporting bias. FLWs may feel compelled to demonstrate comprehensive service delivery and performance, leading to a fear of accurately reporting the truth instead of overestimated numbers. Experts said that in many villages, AWWs and ASHAs report inaccurate numbers of pregnant women, resulting in data mismatches that negatively impact data quality. While numerous dashboards and applications are now available to streamline healthcare services, FLWs report them to be overwhelming. There is a growing need for emphasis on meaningful engagement and support between beneficiaries and healthcare providers, rather than merely on data recording.

Participants also reported shortages of trained personnel, inadequate supportive supervision, and unclear roles across different sectors, all of which contributed to fragmented implementation of maternal nutrition services.

##### Infrastructure and supply chain barriers

Although maternal nutrition services are widely available through public health facilities, respondents identified multiple structural barriers that continue to limit access, particularly in rural and remote areas. Distance to facilities, poor transportation, indirect financial costs and inadequate infrastructure frequently delayed antenatal care attendance and access to maternal nutrition services.

As one of our respondents pointed out, “*poverty and economic hardships prevent many women from accessing maternal nutrition services. Even when services are free, indirect costs like transportation and time off work can become prohibitive*.”

Supply-side challenges further undermined program implementation. These included delayed procurement, stock-outs of essential commodities, inconsistent availability of iron-folic acid (IFA) supplements, poor quality of Take Home Rations (THR), and limited product diversity.

A program expert highlighted: “*There is monotony in the THR products. If you are given the same product for 3 years, anybody can get tired of eating the same thing over and over again. Of course, practically it is not possible that the state produces a hundred varieties of a product, but at least two to three varieties can be done. And then, pregnant women and young mothers can be taught how to cook them creatively and give them to their children. So, in Uttar Pradesh, we are working on making a sweet product as well as a savory. And then we intend to teach them how to cook it with variety.”*

Collectively, these constraints reduced program acceptability and women’s adherence to nutritional interventions.

#### Information systems and program monitoring

##### Weak monitoring systems

Respondents acknowledged recent investments in digital health platforms but emphasized that monitoring systems remain fragmented and insufficiently integrated. While systems such as Family Planning Logistics Management Information System (FPLMIS) and e-Aushadhi have improved logistics management in selected programs, equivalent real-time monitoring mechanisms for maternal nutrition commodities remain absent. Experts also raised concern regarding the prevalent practice of data fudging or manipulation by field data collectors and FLWs, leading to biased reporting of program data.

One of our interlocutors said, “*Suppose I am a frontline functionary and I report data that does not glorify me as a good frontline functionary, then I will never report that! I have seen some bureaucrats in Maharashtra and Rajasthan who have given confidence to community health officials that even if they bring data that presents them poorly in terms of service coverage and quality, they will still reward them for reporting the truth. But most bureaucrats will not want the real data to come out. I think the fear of reporting the truth is also something that probably impacts the quality of the data and monitoring initiatives. I think we need accountability from those in a leadership position rather than the frontline functionaries.”*

Another professional indicated, “*Actionable data comes only when you have a comprehensive strategy in place. So, apps are useful when the systems are robust. There are more than 50 systems of digital information within one state government but no integration. One more app or one more system is not the solution. The solution should emerge from the bottom up. Once that system is strengthened, then your information systems can be used to monitor progress.”*

Participants emphasized that digital technologies alone cannot strengthen maternal nutrition programs unless embedded within well-functioning health systems. Experts argued that strengthening supportive supervision and fostering a culture of transparent reporting would improve data quality more effectively than introducing additional digital applications.

#### Community and sociocultural barriers

##### Sociocultural norms influence the reach and uptake of maternal nutrition services

Stakeholders consistently described social and gender norms as major barriers to women’s access to maternal nutrition services. Limited decision-making autonomy, inadequate family support, food taboos, misconceptions regarding pregnancy and nutrition, and low male involvement reduced women’s ability to adopt recommended nutritional practices.

Participants particularly emphasized the limited engagement of husbands and family members during pregnancy despite their substantial influence over household food allocation and healthcare decisions.

A participant working on maternal health services in Bihar said, *“In Bihar, data showed that 55% of husbands accompanied their wives for the antenatal checkups in villages. But whenever we visited the field, that was not the case. Only in one instance, I saw a husband joining his wife for an antenatal checkup, probably because he came to inquire about something else. I think it was about a cash assistance scheme. I asked him, do you want to understand how the IFA tablet has to be administered to your pregnant wife, why she should focus on her diet and rest and so forth. He was not very interested in that information. It is really important to motivate men to become responsible during a woman’s pregnancy, as husbands, fathers, fathers-in-laws, brothers and sons.”*

Experts also shared that there is a prevalent sentiment of distrust and suspicion toward professionally trained doctors of modern biomedicine in tribal areas. Local communities and families continued to rely on traditional remedies and indigenous practitioners for antenatal care, which ultimately reduced women’s access to and compliance of government-funded maternal nutrition services in India.

##### Limited community ownership of maternal nutrition programs

Experts revealed limited involvement of Self-Help Groups (SHGs), Panchayati Raj Institutions (PRIs), and local communities in promoting maternal nutrition. Existing programs largely rely on frontline workers, with relatively few mechanisms to mobilize community participation or generate local demand for services.

Participants suggested that women who had successfully adopted recommended nutrition practices could serve as community champions to promote positive behavior change during Village Health, Sanitation and Nutrition Days (VHSND). Additionally, panchayat members and block/district officials should be engaged to incorporate health and nutrition services into electoral agendas.

One participant said, “*There will always be one or two women in every community who genuinely follow the advice given and they experience real improvement. If, during the VHSND sessions, such champions are identified and invited to share their experiences, it could make a big impact on others.”*

Overall, stakeholders argued that strengthening community participation and improving male engagement are essential for increasing program uptake, sustaining behavior change and enhancing accountability within maternal nutrition programs.

## Discussion

This study examined maternal nutrition service delivery in India through the perspectives of researchers, program implementers, and policy experts involved in designing, implementing, and evaluating maternal nutrition programs. Rather than evaluating a single intervention, the study provides a systems-level understanding of how maternal nutrition services are organized and where implementation gaps persist despite the existence of comprehensive national policies and programs. By drawing on expert perspectives across multiple levels of the health system, the findings identify the policy, health system, and sociocultural factors that continue to influence women’s access to maternal nutrition services.

Viewed through the WHO Health Systems Framework, the findings demonstrate that barriers to maternal nutrition extend across multiple interconnected health systems building blocks rather than isolated program inadequacies (11). Implementation challenges were evident in the absence of integrated, context-specific maternal nutrition strategies and limited routine program evaluation. Few weaknesses within capacity-building efforts were reflected in inadequate counseling competencies, competing responsibilities, and insufficient supportive supervision for frontline workers. Persistent stock-outs, procurement delays, and limited diversity of nutrition commodities highlighted challenges in access to essential medicines and supplies, while fragmented monitoring systems and concerns regarding data quality underscored broader limitations in health information systems. Collectively, these findings suggest that improving maternal nutrition requires coordinated health system strengthening rather than the introduction of additional stand-alone nutrition interventions.

An important contribution of this study is the distinction it draws between the availability of maternal nutrition services and their effective delivery. India already has a fairly wide array of service delivery and monitoring platforms for maternal nutrition. Services such as IFA and calcium supplementation, deworming, weight monitoring, and take-home rations form the routine package of care for most pregnant women (12, 13). The launch of POSHAN 2.0/Saksham Anganwadi, Anemia Mukt Bharat Abhiyaan (AMB), Surakshit Matritva Aashwasan (SUMAN), and LaQshya (Labor Room Quality Improvement Initiative) reflects the Government of India’s continued commitment to strengthen maternal nutrition and improve the quality of life of pregnant women and newborns through integrated nutrition interventions, quality antenatal and intrapartum care, anemia reduction, and health system strengthening (14–17). Although the qualitative interviews of this study were completed prior to the launch of progressive maternal and child health policies including the SUMAN Roadmap 2030 and AMB 2.0, our findings suggest that the existence of competent maternal nutrition services alone does not guarantee their effective implementation or adequate reach. The government efforts in the direction to strengthen maternal health are heartening but will strongly benefit from robust implementation and operational success across lifecourse. This in turn is shaped by local health system capacity, frontline worker support, supply chain reliability, and the broader social context within which women make nutritional decisions. These findings reinforce growing evidence that implementation quality, rather than program availability alone, determines the effectiveness of maternal nutrition interventions (18).

India is a highly diverse country, where each state differs from another in terms of community beliefs, food habits, availability of nutritious foods, health system capacity, and cultural expectations surrounding pregnancy and child birth. Therefore, a universal approach cannot adequately capture the local and cultural nuances of how families take healthcare decisions or how women access services. Several experts felt that existing programs would benefit from being more sensitive to local realities and cultural contexts, whether through the way counseling is delivered or in how communities are engaged. Recent studies from the region have made similar observations, noting that interventions work better when they reflect the everyday constraints and cultural practices of the communities they aim to serve (18).

The interviews also highlight the central role of FLWs in translating national nutrition policies into routine practice. Although ASHAs, ANMs, and Anganwadi Workers remain the primary interface between the health system and pregnant women, respondents consistently distinguished between delivering health messages and providing effective nutrition counseling. Heavy workloads, competing program responsibilities, and limited training in behavior change communication constrained their ability to provide individualized counseling, address misconceptions regarding iron-folic acid supplementation, and support sustained dietary behavior change. Similar observations have been reported across South Asia, where frontline workers frequently possess adequate technical knowledge but lack the time, supportive supervision, and communication skills necessary to deliver person-centered nutrition counseling (19, 20).

Several respondents discussed supply-related issues as practical hurdles that tend to slow down service delivery. In some places, the supply of supplements, equipment and medicines take a long time to arrive, and in a few remote areas there are periods when essential items run short. These gaps, even if temporary, interrupt how smoothly services can be delivered. Earlier studies examining IFA and micronutrient supplies in rural India noted the same concerns and pointed out that simple, real-time tracking tools help program staff know when stocks are running low and take timely action (21). Some interviewees also reminded us that the government already uses efficient logistics systems in other programs, for instance, family planning, and felt that similar approaches could be adapted for nutrition items as well. The challenges related to stock-outs, procurement delays, and inconsistent availability of nutrition commodities reflect broader weaknesses in access to essential medicines and technologies, another core component of the WHO Health Systems Framework.

Another notable finding is the limited integration of nutrition across the continuum of women’s health. Participants consistently identified the preconception period as a missed opportunity for addressing micronutrient deficiencies and improving women’s nutritional status before pregnancy. Current maternal nutrition programs in India remain predominantly pregnancy-focused despite increasing recognition that nutritional status before conception substantially influences maternal and neonatal outcomes. This finding supports recent global recommendations advocating a life-course approach to women’s nutrition that extends beyond antenatal care to include adolescence and preconception health (18, 22).

Supply chain challenges emerged as another important implementation bottleneck. Respondents described recurrent delays in procurement, inconsistent availability of IFA supplements and THR, and limited mechanisms for monitoring commodity availability at service delivery points. While digital logistics platforms including the FPLMIS have strengthened commodity management in other national programs, participants highlighted the absence of comparable real-time systems for maternal nutrition commodities. These findings suggest that strengthening logistics management and adapting existing digital supply chain innovations may improve program continuity while reducing stock-outs and service interruptions (21).

The study further highlights that although digital health systems are effective in streamlining services and medical records, they alone cannot strengthen program implementation without corresponding investments in health governance and accountability. Participants expressed concerns regarding fragmented information systems, duplication of reporting requirements, and incentives that favor reporting program performance over reporting program realities. Consistent with previous studies on digital health implementation in India, respondents argued that improving data quality depends less on introducing additional applications than on creating supportive systems that encourage accurate reporting and facilitate the routine use of data for decision-making (22). This finding contributes to ongoing implementation science debates by emphasizing that digital innovations should strengthen, rather than replace, underlying health system functions.

Beyond health system constraints, the findings demonstrate that maternal nutrition practices remain deeply embedded within broader social and gender norms. Women’s dietary practices and healthcare decisions continue to be influenced by husbands, mothers-in-law, household food allocation, and longstanding cultural beliefs surrounding pregnancy and nutrition (23). Respondents also emphasized the underutilized role of Self-Help Groups, Panchayati Raj Institutions, and community champions in promoting nutrition awareness and generating local demand for services. These findings reinforce existing evidence demonstrating that improving maternal nutrition requires community-based approaches that engage families and local institutions alongside formal health services (24, 25).

Overall, this study contributes to the existing scholarship on maternal nutrition by moving beyond descriptions of individual program barriers to provide a comprehensive health systems perspective on maternal nutrition service delivery in India. Through expert interviews from across India, the study demonstrates that despite government efforts to provide structured and universal care focused on maternal nutrition, persistent implementation gaps arise from the interaction of governance, workforce capacity, supply systems, information systems, and diverse sociocultural contexts. The findings underscore that existing maternal nutrition strategies should prioritize strengthening implementation quality, investing in frontline counseling capacity, integrating nutrition across the continuum of women’s health, improving supply chain and information systems, and fostering stronger community engagement instead of introducing additional schemes at the state and national level. Such system-oriented approaches are essential for ensuring that existing maternal nutrition programs translate into meaningful improvements in women’s nutritional health and pregnancy outcomes across diverse settings in India.

## Limitations

This study included expert perspectives from diverse regions of India, enabling the identification of socioeconomic and systemic barriers to maternal nutrition service delivery across varied contexts. Although in-depth interviews were conducted using a semi-structured guide, the findings rely on self-reported information, and it was not possible to clearly ascertain the extent to which participants may have over- or under-reported issues related to service delivery challenges. The qualitative component included interviews with 15 experts. Although thematic saturation was achieved and participants represented diverse policy, implementation, and research perspectives, the relatively small sample may not capture the full range of views across India’s heterogeneous maternal nutrition landscape. Consequently, the study did not include direct interviews with frontline health workers or beneficiaries; therefore, the results reflect system-level perspectives rather than the lived experiences of FLWs and pregnant women.

## Conclusion

This study brings together expert perspectives on why maternal nutrition services do not always reach pregnant women as intended. While India has several programs and platforms to enhance maternal nutrition, discussions with experts highlighted several implementation challenges across the health system, program delivery, and community contexts, impacting the delivery and uptake of maternal nutrition services. At the system level, policy efforts could be directed toward developing an integrated maternal nutrition strategy that addresses the continuum of care, from preconception to pregnancy, and considers the dual burden of malnutrition. This includes strengthening multisectoral coordination, improving monitoring and data quality, and providing supportive supervision to align program implementation with community needs and cultural contexts. At the program level, strengthening implementation could involve investing in frontline workforce capacity, improving nutrition counseling and behavior change communication, reducing reporting burdens on FLWs, and integrating preconception counseling within existing service platforms. At the community level, meaningful engagement of families, particularly husbands and other household decision-makers, together with Self-Help Groups, Panchayati Raj Institutions, local leaders, and community champions may strengthen community ownership, support sustained behavior change, and subsequently improve the effectiveness and acceptability of maternal nutrition services. Investing in further research including beneficiaries and FLWs could provide additional insights to strengthen context-specific maternal nutrition services. Overall, strengthening the implementation and monitoring of existing maternal nutrition programs while accounting for local health systems and sociocultural contexts may offer opportunities for improving the reach and effectiveness of maternal nutrition services in India.

## Data Availability

The raw data supporting the conclusions of this article will be made available by the authors, without undue reservation.

## Glossary

IFA: Iron Folic Acid
Ca: Calcium
FA: Folic Acid
THR: Take Home Ration
HCM: Hot Cooked Meal
AWC: Anganwadi Centre
ANM: Auxiliary Nurse Midwife
ASHA: Accredited Social Health Activist
AWW: Angan Wadi Worker
FLW: Front Line Workers
ICDS: Integrated Child Development Scheme
NHM: National Health Mission
IEC: Information, Education and Communication
SBCC: Social Behavior Change and Communication
MO: Medical Officer
AWHs: Anganwadi Helpers
NFHS: National Family Health Survey
PRI: Panchayati Raj Institution
PW&LM: Pregnant Women and Lactating mothers
SHG: Self Help Group
SAM: Severely Acute Malnourished
GOI: Government of India
VHSNC: Village Health Sanitation and Nutrition Committee
