# Supplementary material for: Assessing maternal nutrition policy and programmatic landscape in India: a qualitative study
Source: Front Nutr. 2026 Oct 7;13:1831098. doi: 10.3389/fnut.2026.1831098 (PMC13683634; doi:10.3389/fnut.2026.1831098)
Supplement: Supplementary file 1 [file Supplementary_file_1.docx]

**Supplementary File 1: Interview Guide**

 Introduction- Hello! We are conducting a project titled, "Maternal Nutrition Gaps in India: Exploring Reasons for Service Delivery Discrepancies and Utilization Barriers - A Mix-method Study" where we intend to examine the barriers and facilitators of different maternal nutrition services in India. I am reaching out to you because of your esteemed expertise in maternal nutrition which will greatly contribute to this research. We ensure that your responses will be anonymous and confidentiality is maintained. Please feel free to share any information that you believe will be of interest to this research.

Opening Question- Can you share your experience in the area of nutrition, focusing on Maternal Nutrition?

What are the current gaps in service delivery and utilization of maternal nutrition services in India?

A. Access and utilisation of services

1.	Are pregnant women able to access health and nutrition services?

2.	What gaps exist in access to services?

3.	How can these gaps be addressed?

4.	In which areas, do you think utilization of maternal nutrition services is low, and what are the reasons behind it?

5.	How can the utilization of services be improved?

B. Supplements and Equipment

1.	Do you think there are gaps in the procurement of nutrition supplies?

2.	If yes, then what are the gaps in the supply chain management of supplements like IFA, Ca, and Deworming tabs and how can these gaps be addressed?

3.	How can supply chain efficiency be improved for better service delivery?

C. Skilled and motivated workforce/ Capacity Building

1.	Do you think there are gaps in the capacity building of the workforce?

2.	What gaps exist in the capacity of healthcare professionals and what training interventions are necessary to bridge these gaps?

D. Demand for services

1.	Do you think there is a need to increase demand for services via counselling, campaigns, health education, and messaging?

2.	What gaps exist in demand generation, and how can they be worked upon?

3.	What new interventions could be planned for generating more demand for nutrition services?

Data recording and quality

1.	Are there gaps in data reporting, and how can data reporting be improved?

2.	Do we have quality data for nutrition?

3.	How can data quality be improved?

E. Policy

What can be the policy-level changes required in the existing programmes to improve maternal nutrition?

Closing Remarks- Is there anything more you would like to add?

Thank you for your time.

**Supplementary Table 1: State-Level Nutrition-Specific and Nutrition-Sensitive Initiatives for Women, Children, and Adolescents in India**

| **State** | **Department** | **Name of Initiative** | **Intervention** | **Targeted Groups** | **Geography** |
| --- | --- | --- | --- | --- | --- |
| **Odisha** | Government of Odisha | Separate Budget for Nutrition programmes (nutrition-specific and sensitive). | Dedicated nutrition-specific and nutrition-sensitive budget allocation to strengthen multisectoral nutrition actions. | Women, Children, adolescents | Entire State |
|  | Women & Child Development | Mukhymantri Samproona Pushti Yojana (MSPY) | Additional Take Home Ration (THR) in the form of nutrient rich food products, including millets and additional eggs | PW & LW, Adolescent girls | Entire State |
|  |  | MAMATA | Conditional cash transfer of Rs 3000- Maternity benefit scheme | PW and LW above 19 yrs of age for first two live births | Entire State |
|  |  | Providing varied THR menu | Diversification of THR formulations to improve acceptability and dietary diversity. | PW, lactating women, Children and adolescents | Entire State |
|  | Agriculture | Odisha Millets Mission (OMM) | Promotion of millet production and consumption to improve nutrition security in tribal regions. | Entire tribal population of 19 districts. | 19 districts |
| **Haryana** | Women and Child Development | Mukhya Mantri Doodh Uphaar Yojna | Distribution of fortified skimmed milk (Vitamin D3) to improve maternal and Child nutrition. | PW, Lactating women and 1-3 year Children | Entire State |
| **Gujarat** | Women and Child Development | POSHAN SUDHA | Spot Feeding Scheme: Provision of full meals, IFA, calcium supplementation, and nutrition education at AWCs. | PW & Lactating women | 14 Tribal Districts |
|  | Women & Child Development | Mukhya Mantri Matru Shakti Yojana | Provision of nutrition kits comprising of 1 Litre of Groundnut oil, 1 Kg Tuver Dal, & 2 Kg Chana | PW & LW, Adolescent girls | Entire State |
|  | Women & Child Development | Providing varied THR menu | Diversification of THR formulations to improve acceptability and dietary diversity. | PW, lactating women, Children and adolescents | Entire State |
|  | Women & Child Development | DOODH SANJIVANI SCHEME | Distribution of fortified flavored milk to address micronutrient deficiencies. | Children, PW & LW | 20 districts |
|  | Women & Child Development and AYUSH | Fortified ready to cook pre-mix | AYUSH-enriched premix to improve appetite, nutrient absorption, and maternal health outcomes. | PW, LW | Entire State |
|  | Women and Child Development | Double Fortified salt | Monthly provision of iron- and iodine-fortified salt to address micronutrient deficiencies. | PW, LW | Entire State |
|  | Health and Family Welfare | Namoshree Scheme | Providing cash incentives for improved health and nutrition to pregnant and lactating women. | PW, LW | Entire State |
| **Madhya Pradesh** | Women & Child Development | UDITA Yojana | The scheme prepares activities for adolescent girls by creating a platform to discuss issues related to nutrition and health of adolescent girls. | All adolescent girls and women between 18 to 49 years will be beneficiaries | Entire State |
|  | Women and Child Development | Millet-based THR | Introduction of millet-based THR to improve dietary diversity. | Children aged 6 months to 3 years, PW, LW | Dindori District |
|  | Women & Child Development | Providing varied THR menu | Diversification of THR formulations to improve acceptability and dietary diversity. | PW, lactating women, Children and adolescents | Entire State |
| **Tamil Nadu** | Health and Family welfare Department | Dr. Muthulakshmi Maternity Benefit Scheme | Financial assistance and nutrition kits to improve maternal health outcomes. | PW, LW | Entire State |
|  | Women & Child Development | Providing varied THR menu | Diversification of THR formulations to improve acceptability and dietary diversity. | PW, lactating women, Children and adolescents | Entire State |
| **Maharashtra** | Women and Child Development | Atal Bihari Vajpayee Bal Arogya Poshan Yojana | Provision of hot cooked meals, including doorstep delivery during late pregnancy and early postnatal period | PW and LW above 19 yrs | 16 districts: 85 ICDS projects in scheduled areas; covers 16,030 AWCs and 2,013 mini AWCs |
| **Chhattisgarh** | Women and Child Development | Chief Minister’s Nutrition Campaign | Provision of fortified THR and hot cooked meals, including millet integration. | Children 0-6 yrs,PW, LW | Entire State |
|  | Women and Child Development | Millet-based THR | Introduction of millet-based THR to improve dietary diversity. | Children 0-6 yrs,PW, LW | 2 Districts -Kanker, Raigarh |
|  | Women and Child Development | Mahtari Jatan Yojana | Provision of HCM, IFA, and THR to pregnant women. | PW | 28 Districts |
|  | Labour Department | Minimata Mahtari Jatan Yojana | Cash assistance to improve nutrition among pregnant women in the unorganized labor sector. | PW working as laborers in the unorganized sector of building and construction work or whose husbands work as laborers | Entire State |
| **Rajasthan** | Women and Child development | Rajasthan Indira Gandhi Matritva Poshan Yojana | Conditional cash assistance to support maternal nutrition during pregnancy. | PW | Entire State |
|  | Department of WCD, Health and TAD (Tribal Area Development Department) | PUKAR | Community-based outreach through weekly visits providing nutrition counseling and ICDS linkage. | PW, LW | Bikaner District |
| **Andhra Pradesh** | Women, Child, Disabled and Senior Citizens Department | Anna Amrutha Hastham | Integrated nutrition intervention including HCM, eggs, milk, supplements, and monitoring. | PW, LW | Entire State |
|  | Women and Child Development | YSR Sampoorna Poshana Plus scheme | Enhanced THR and HCM with millet-based and energy-dense foods | PW, LW, Children 6-36 months Children 36-72 months | 77 Scheduled and Tribal Sub-Plan Mandals |
|  | Women Development and Child Welfare | Chandranna Giri POSHANA Kendra | Provision of ready-to-cook and ready-to-eat millet-based foods for nutritionally vulnerable populations, complemented by hot cooked meals for Children and women residing in remote and hilly tribal habitations. | PW, LW, and Children from 6 months to 6 years are covered in this scheme. | Tribal Areas |
|  | Women & Child Development | Providing varied THR menu | Providing varied THR menu options to boost acceptability and provide additional choices among recipients | PW, lactating women, Children and adolescents | Entire State |
| **Telangana** | Women, Child, Disabled and  Senior Citizens Department | Arogya Lakshmi | Comprehensive maternal nutrition package including HCM, supplements, and intensified follow-up. | PW , LW | Entire State |
|  | Tribal Welfare and Women and Child Welfare departments | Giri Poshana Yojana | Provision of three ready-to-cook formulations (multi-grain cereal, jowar meal, and multi-grain sweet meal) and three ready-to-eat products (peanut–sesame chikki, peanut–fried gram chikki, and jowar bites | Children, adolescent, PW, LW | Tribal Areas |
|  | Women & Child Development | Providing varied THR menu | Diversification of THR formulations to improve acceptability and dietary diversity. | PW, lactating women, Children and adolescents | Entire State |
| **Karnataka** | Women & Child Development | Mathrupoorna Yojana | Facility-based HCM with mandatory IFA intake, deworming, and nutrition counseling. | PW, LW | Entire State |
|  | Women & Child Development | Providing varied THR menu | Diversification of THR formulations to improve acceptability and dietary diversity. | PW, lactating women, Children and adolescents | Entire State |
| **Kerala** | Women & Child Development | Fortified THR | Locally produced, multi-micronutrient fortified THR to address maternal and Child undernutrition. | PW, Lactating women and Children | Entire State |
| **Chandigarh** | Women and Child Development | Millet-based THR | Introduction of millet-based THR to improve dietary diversity. | Children, pregnant women, lactating mothers, and adolescent girls, | Entire UT |
|  | Women & Child Development | Providing varied THR menu | Diversification of THR formulations to improve acceptability and dietary diversity. | PW, lactating women, Children and adolescents | Entire State |
| **Bihar** | Providing eggs and doubt fortified salt | Providing eggs and doubt fortified salt | Introduction of eggs and double-fortified salt to improve protein and micronutrient intake. | PW, Children | Eggs in all districts Double fortified Salt in Tirhut District |
| **Uttarakhand** | Women & Child Development | Providing varied THR menu | Diversification of THR formulations to improve acceptability and dietary diversity. | PW, lactating women, Children and adolescents | Entire State |
| **Himachal Pradesh** | Women & Child Development | Providing varied THR menu | Diversification of THR formulations to improve acceptability and dietary diversity. | PW, lactating women, Children and adolescents | Entire State |
|  | National Health Mission, Women and Child Development, and Department of Elementary Education | Mukhya Mantri Bal Suposhan Yojana | Provision of supplementary nutrition with animal-source protein (*Purak Aahar*), complemented by community engagement activities promoting locally available nutritious foods. Mobile AV-enabled outreach and village-level discussions strengthen awareness on balanced diets, IFA compliance, and nutrition myths. | The scheme not only targets the Children, PW & LW, but also reaches out to community members. | Entire State |
| **Manipur** | Women & Child Development | Providing varied THR menu | Diversification of THR formulations to improve acceptability and dietary diversity. | PW, lactating women, Children and adolescents | Entire State |
| **Mizoram** | Women & Child Development | Providing varied THR menu | Diversification of THR formulations to improve acceptability and dietary diversity. | PW, lactating women, Children and adolescents | Entire State |
| **Tripura** | Social Welfare | Mukhyamantri Matru Pushti Uphaar | Nutrition kits and conditional cash transfers linked with antenatal check-ups. | PW, LW | Entire State |
| **Jammu & Kashmir** | State Health Department in convergence with the ICDS Department | HCM | Provision of hot cooked meals | PW | Entire State |
| **Andaman and Nicobar Islands:** | Social Welfare | Suposhan Diwas | Community mobilization and nutrition promotion activities integrated with growth monitoring. | 0-6years), pregnant women, lactating mothers and adolescent girls | 720 Anganwadi Centres |
| **Assam** | Introduced by NGO – NEDSF with UNICEF in collaboration with Department of Social Welfare | Matri Amrit Ahar | Community-based nutrition ceremonies promoting maternal nutrition and institutional delivery. | PW | 5 districts- Morigaon, Kamrup, Barpeta, Goalpara and Darrang |
|  | Health and Family Welfare | Wage Compensation Scheme for Pregnant Women of Tea Garden Areas of Assam | Cash compensation to protect livelihoods and nutrition of pregnant women in tea garden areas. | All PW residing in Tea garden areas are eligible for the scheme. The pregnant women may be Temporary worker, permanent worker or non worker – all are eligible. | Entire State |
| **Sikkim** | Health Care Human service & Family Welfare Department | Proud Mothers Scheme | Provision of monetary assistance to pregnant women (first and second live births) and their Children up to six years to improve access to healthcare during and after delivery | All pregnant women residing | Entire State |
| **Meghalaya** | Early Childhood Development (ECD) Mission in collaboration with the department social welfare and Meghalaya State Rural Livelihood Society. | Introduction of boiled eggs | Supplementation of ICDS meals with boiled eggs to improve protein intake. | PW, LW, Children | 50 Model Anganwadi Centers (AWC’s)- The supply of eggs will eventually be rolled out across all Anganwadi centers and uncovered villages in a phased manner |
| **Daman and Diu** |  | Awareness generation | Effective use of platforms like “Gram Sabha”, “Construction Sites”, “Otalni Varta” with SHGs member to create awareness about POSHAN ABHIYAAN. | All residents of UT | Entire UT |

***Supplementary Figure 1: Key Ministerial Platforms for Delivering Maternal and Child Health and Nutrition Services***
***Supplementary Figure 2: Proposed Integration of Nutrition Interventions into Existing Government Platforms***

**Figure 3: NFHS 4 & 5 State-wise prevalence of underweight among pregnant women in 1st trimester**

**Figure 4: NFHS-5 State-wise prevalence of Underweight (%) among pregnant women in 1st trimester**

**Figure 5: NFHS 4 & 5 State-wise prevalence of overweight/obese (%) among pregnant women in 1st trimester**

**Figure 6: NFHS-5 State-wise prevalence of overweight/obese (%) among pregnant women in 1st trimester**
